# Differential autophagy cargo engagement in amyloid-β-induced neuronal toxicity

**DOI:** 10.3389/fnins.2026.1913175

**Published:** 2026-09-24

**Authors:** Danielle Brink, Rensu Theart, Benjamin Loos

**Affiliations:** 1 Department of Physiological Sciences, Stellenbosch University, Stellenbosch, South Africa; 2 Department of Electric and Electronic Engineering, Stellenbosch University, Stellenbosch, South Africa

**Keywords:** Alzheimer's disease, amyloid, autophagy, fluorescence, neurodegenerative disease

## Abstract

Neurodegenerative diseases are characterized by progressive neuronal loss and the accumulation of misfolded proteins. In Alzheimer's disease, several pathogenic protein species, including amyloid precursor protein, amyloid beta, tau, phosphorylated tau, and alpha-synuclein, are known substrates of macroautophagy. However, the extent to which macroautophagy selectively engages these proteins for degradation under conditions of proteotoxic stress remains poorly understood. Here, the contribution of specifically macroautophagy to the degradation of amyloid precursor protein, amyloid beta, tau, phosphorylated tau, and alpha-synuclein was investigated using an amyloid precursor protein overexpression model. Mouse neuroblastoma N2A cells stably expressing the human Swedish amyloid precursor protein mutation were treated with butyric acid for 24 and 48 h to produce increasing proteotoxicity and molecular pathology. Autophagic flux was assessed using bafilomycin A1 and protein abundance quantified by western blotting. Confocal microscopy and colocalization analysis was performed to examine intracellular protein localization and recruitment to the autophagy compartment. Amyloid precursor protein overexpression and autophagosome/lysosome fusion inhibition elicited distinct protein-specific responses, with phosphorylated tau showing highest accumulation and autophagic recruitment. In contrast, tau and amyloid precursor protein exhibited moderate increases, while alpha-synuclein and amyloid beta showed differential and limited responses upon macroautophagy inhibition. Collectively, these findings suggest that the autophagy molecular machinery does not engage uniformly with Alzheimer's disease-associated proteins but may be characterized by a highly differential cargo engagement under amyloid precursor protein-driven proteotoxic stress. Phosphorylated tau emerged as the earliest and most prominent protein to be target to the autophagy compartment, highlighting its potential importance in disease progression and therapeutic intervention.

## Introduction

Neurodegenerative diseases are an increasing global health concern, characterized by the progressive and irreversible loss of neurons in specific brain regions, often associated with the accumulation of misfolded proteins (Dugger and Dickson, 2017). Among neurodegeneration, Alzheimer's disease (AD) is the most prevalent, initially presenting with memory loss and cognitive decline that progressively impairs essential neurobehavioral functions (Schultz et al., 2004). The pathological hallmarks of AD include extra- and intracellular amyloid-beta (Aβ) deposition and intracellular neurofibrillary tangle formation through hyperphosphorylated tau (Wisniewski et al., 1997). Currently affecting over 50 million people worldwide, AD prevalence is projected to triple by 2050 (Patterson, 2018), with a substantial burden expected to increase in low- and middle-income countries (Nandi et al., 2022), making this a particular health concern for the global south. Despite its growing impact, effective disease-modifying treatment remains limited, highlighting the need for improved understanding of underlying mechanisms that drive AD pathogenesis.

Autophagy is a key intracellular degradation system essential for maintaining neuronal proteostasis and cell survival. It comprises three main pathways: macroautophagy (MA), chaperone-mediated autophagy (CMA), and microautophagy, which collectively form a dynamic and adaptive network for protein quality control (Cuervo, 2004; Assaye and Gizaw, 2022). MA is responsible for the degradation of bulk cytoplasmic content, including long-lived proteins, through the formation of autophagosomes that fuse with lysosomes to enable cargo degradation and recycling (Wen and Klionsky, 2016). In contrast, CMA is a highly selective pathway that targets proteins containing a KFERQ-like motif (Dice, 1990), facilitating their direct translocation into lysosomes via the receptor lysosome-associated membrane protein type 2A (LAMP2A) (Kaushik and Cuervo, 2018), which regulates the rate-limiting step of this process (Arias, 2017). Given that AD is characterized by the accumulation of toxic protein aggregates (Ruz et al., 2020), autophagy has emerged as a critical pathway impacting disease progression and a promising therapeutic target. Key proteins implicated in AD, including amyloid precursor protein (APP), Aβ, tau, and phosphorylated tau (p-tau), are known substrates of MA (Nixon, 2007; Hamano et al., 2021). Additionally, alpha-synuclein (α-syn), a protein primarily associated with Parkinson's disease, is also degraded via MA (Webb et al., 2003) and has increasingly been implicated in AD. Notably, α-syn-containing Lewy bodies are present in a significant proportion of both sporadic and familial AD cases (Lippa et al., 1998; Hamilton, 2000; Parkkinen et al., 2003), suggesting cross talk and a potential role in disease progression.

Despite evidence that multiple AD-related proteins are degraded by MA, it remains uncertain whether this pathway preferentially targets specific substrates under conditions of proteotoxic stress. Understanding the selectivity and sensitivity of proteinaceous cargo to MA is therefore critical, as it may reveal proteins that are most effectively cleared by the cell and may contribute most significantly to cellular dysfunction upon accumulation. This study therefore aimed to investigate the relative contribution, sensitivity and cargo engagement profile of MA in the degradation of specifically APP, Aβ, α-syn, tau, and p-tau under conditions of amyloid-induced toxicity.

## Materials and methods

### Cell culture protocol

Mouse neuroblastoma cells over-expressing the human Swedish APP mutation (N2a-APP) induced via butyric acid exposure (BA) (Thinakaran et al., 1996; Luo et al., 2002; Ntsapi and Loos, 2021) were acquired from Professor Sangram Sisodia (Thinakaran et al., 1996) (Department of Neurobiology, University of Chicago, USA). N2a-APP were cultured in Dulbecco's Modified Eagle's Medium (DMEM, ThermoFisher, #41965062). DMEM was supplemented with 10 % fetal bovine serum (Biocom Africa, #FBS-GI-12A), and 1 % Penicillin-Streptomycin (ThermoFisher, #15070063); 100 U/mL of penicillin and 100 μg/mL of streptomycin and maintained at 37 °C in a humidified environment in the presence of 5 % CO_2_. N2a-APP were sub-cultured when 70-90 % cell confluence was reached using trypsin (ThermoFisher, #25200072) for 5 min at 37 °C to cause detachment of adherent cells from the flask. Following trypsinisation, DMEM was added in a 2:1 ratio and cells were collected into a 15 mL falcon tube (Bio-Smart, #70025). Cells were centrifuged (Eppendorf Centrifuge 5804R) at 1,500 rpm for 3 min at room temperature. The supernatant was discarded, and cells were resuspended in fresh 1mL DMEM. Cells were seeded into T25 culturing flasks (Bio-Smart, #70025), 48-well cell culture plates (Bio-Smart, #30048), or 8-well chamber dish with cover glass (Nunc Lab-Tek, #155409), depending on experimental requirements.

### Treatment conditions

To induce endogenous APP overproduction, N2a-APP cells were cultured in the presence of 5 mM BA (Sigma-Aldrich, B103500) for 24h, and 48 h, respectively, with each treatment group being compared to an untreated, control group (Thinakaran et al., 1996; Luo et al., 2002; Ntsapi and Loos, 2021). To ascertain the role of macroautophagy activity, i.e., autophagy flux, during APP overexpression, cells were treated with saturating concentrations of bafilomycin A1 (BafA1, LKT labs, B0025), a selective inhibitor that inhibits autophagosome/lysosome fusion (Mauvezin and Neufeld, 2015). BafA1 was made up using filtered DMSO (Sigma-Aldrich, #276855-1L). Cells were treated with BA for 24 h and 48 h, respectively, followed by a 2 h incubation in the presence (B) or absence (C) of 400 nM BafA1. The treatment groups were labeled as the following: Control 0h (C0), Control 24h (C24), Control 48h (C48), Bafilomycin 0h (B0), Bafilomycin 24h (B24), Bafilomycin 48h (B48). Following treatment, cells were either harvested for protein analysis by western blotting or prepared for confocal imaging.

### Western blot analysis

For protein extraction, culture medium was removed and cells were rinsed three times with cold 1X Phosphate Buffered Saline (PBS, Sigma Aldrich; #P4417-100tab). 100 μL of RIPA lysis buffer (50 mM Tris-HCl, 1 mM sodium fluoride, 0.25% sodium-deoxycholate, 150 mM NaCl, 1% Triton-X, 1 mM EDTA, 1 mM PMSF, 1 μg/mL leupeptin, 1 mM Na3VO4, 1 ug/mL aprotonin, 1 ug/mL benzamidine, 10 ug/mL pepstatin) was added to the cells and incubated for 10 min on ice. Next, cells were scraped off using a cell scraper (WhiteSci, #71000). Lysates were collected, vortexed and left on ice for 1 h. Next, lysates were centrifuged at 8,000 rpm for 10 min at 4°C (Megafuge 8R Centrifuge, Thermo Scientific). Protein concentration was determined using a Bradford assay as previously described (Bradford, 1976). A working solution of Laemmli's loading buffer (0.8 mL glycerol (Merck, #1045422), 1 mL Tris-HCl (0.5 M; pH 6.8), 1.6 mL 10 % sodium dodecyl sulfate (SDS, Sigma-Aldrich,74255-250G), 0.4 mL of 0.05 % Bromophenol blue (Merck, #1041675), 3.8 mL deionized H_2_O) was prepared by mixing Laemmli's loading buffer with 15 % of β-mecaptoethanol (Sigma-Aldrich, M3148-100mL). 50 μg of protein sample was prepared for subsequent loading with Laemmli's loading buffer in a 2:1 ratio. Samples were boiled at 95 °C for 5 min. Proteins were separated on a 12% Fast Cast TGX Stain-Free SDS-PAGE gel (Lasec; #BBRD1610185) and electro-transferred onto a polyvinylidene difluoride (PVDF) membrane (Merck; #GE10600021). Nonspecific binding sites were blocked by incubating the PVDF membranes in Everyblot Blocking Buffer (Bio-Rad; #12010020) for 15 min. The membranes were then incubated overnight at 4 °C in their respective primary antibodies (see Table 1), whereafter membranes were rinsed three times with TBS-T (137 mM NaCl, 20 mM Tris, 0.1% Tween-20, pH 7.6) for 5 min before being incubated with their respective horseradish peroxidase-labeled secondary antibodies (see Table 1) for 1 h at room temperature. Protein bands were visualized using Clarity Western ECL substrate (Lasec; BBRD1705761) and the Bio-Rad ChemiDoc MP Imaging System. The Bio-Rad Image Lab (version 6.1.0) software was used to measure the intensity of the protein bands.

**Table 1:** Antibodies and dilutions employed for western blotting.

Primary antibodies					
Antibody	Company	Catalog #	Host species	Size (kDa)	Dilution (μL)
**APP**	Abcam	ab32136	Rabbit	100	1:1000
**Aβ**	Santa-Cruz	sc-23865	Mouse	4-46	1:1000
**α-syn**	Cell Signaling	51510s	Rabbit	100	1:909
**Tau**	Cell Signaling	4019s	Mouse	40-70	1:5000
**P-tau**	Cell Signaling	11837s	Rabbit	50-80	1:1000
**LC3B**	Cell Signaling	2775s	Rabbit	14-16	1:3000
**LAMP2a**	ThermoFisher	PA1-655	Rabbit	46	1:1000
**HSC-70**	ThermoFisher	MA3-014	Mouse	70	1:2000

### Immunofluorescence

N2a-APP cells were seeded at a density of 6 x 10^3^ cells/chamber in 8-chamber dishes and incubated for 12 h. Following the treatment protocol, media was discarded, and cells were fixed for 12 min at 37 °C in 4 % paraformaldehyde (Electron Microscopy Sciences, 15700-1L) prepared in pre-warmed DMEM. Cells were gently washed three times in PBS and then permeabilised by 0.15 % Triton-X (Sigma Aldrich; T8787-100mL) for 6 min. Cells were then rinsed three times PBS before being treated with 1% BSA for 30 min as a blocking step. Next, the cells were incubated overnight at 4 °C with a master mix containing LC3B and one of the following primary antibodies: APP, Aβ, α-syn, tau, or p-tau (see Table 2). After three washes in PBS, the cells were incubated with their respective secondary antibody for 90 min at room temperature (see Table 2). Cells were washed three times with PBS and nuclei counter-stained with Hoechst (ThermoFisher, H3569) solution for 10 min. After three washes in PBS, 1 drop of Fluoromount Aqueous Mounting Medium (Sigma-Aldrich, #F4680-25mL) were added to each well. Micrographs were acquired using the Carl Zeiss LSM780 with ELYRA PS.1 microscope using a 100x oil immersion objective lens. Z-stacks were processed and colocalization analysis was performed using the Java-based, open-source software Fiji (Image J).

**Table 2:** Antibodies and dilutions employed for immunofluorescence.

Horse radish peroxidase secondary antibodies					
Secondary antibody		Company	Catalog #	Host species	Dilution (μL)
**Anti-rabbit IgG HRP**		Cell Signaling	7074s	Goat	1:10 000
**Anti-mouse IgG HRP**		Cell Signaling	7076s	Horse	1:10 000
Primary antibodies					
Primary antibody	Company	Catalog #	Host species	Size (kDa)	Dilution (μL)
APP	Abcam	ab32136	Rabbit	100	1:200
Aβ	Santa-Cruz	sc-23865	Mouse	4-46	1:200
α-syn	Cell Signaling	51510s	Rabbit	100	1:200
Tau	Cell Signaling	4019s	Mouse	40-70	1:100
P-tau	Cell Signaling	11837s	Rabbit	50-80	1:100
LC3B	Cell Signaling	2775s	Rabbit	14-16	1:200
LC3B	Cell Signaling	83506s	Mouse	14-16	1:200
Fluorophore secondary antibodies					
Secondary antibody	Fluorophore	Company	Catalog #	Host species	Dilution (μL)
**Anti-rabbit**	Alexa 488	ThermoFisher	A32790	Donkey	1:1000
**Anti-mouse**	Alexa 488	ThermoFisher	A21202	Donkey	1:1000
**Anti-rabbit**	Alexa 568	ThermoFisher	A10042	Donkey	1:1000
**Anti-mouse**	Alexa 568	ThermoFisher	A10037	Donkey	1:1000
**Actin-Phalloidin**	Alexa 633	ThermoFisher	A22284	N/A	1:200
Fluorophore properties					
Fluorophore dye	Absorbance wavelength (nm)		Emission wavelength (nm)		Visible color
Hoechst 33342	350		461		Blue
Alexa Fluor 488	490		525		Green
Alexa Fluor 568	579		603		Red
Alexa Fluor 633	632		647		Far-red

To quantify the degree of colocalization, thresholded Manders' coefficients were determined as previously described (Adler and Parmryd, 2010). Micrograph stacks of the green (LC3-protein), magenta (actin filaments), and red (candidate protein of interest) channel were deconvolved separately using the repective theoretical PSF generated with the PSF Generator, Fiji (Java 1.8.0_172) DeconvolutionLab2 plugin with the Richardson–Lucy Total Variation (RLTV) algorithm, 8 iterations, and a regularization parameter of 1.0 × 10^−5^. Deconvolved image stacks were background corrected across the complete z-stack using Fiji's rolling-ball background subtraction with a radius of 5 pixels. An independent three-dimensional cytoplasm mask was generated with the magenta actin filaments micrograph stack serving as estimate for the whole cell region, while the blue channel (Hoechst) micrograph stack was used to identify the nuclear region. Before thresholding, the Sigma Filter Plus was applied using a radius of 2 pixels, pixels within 2 standard deviations, a minimum pixel fraction of 0.2, and outlier-aware processing. The micrograph stacks in the magenta channel were thresholded with the Li method and stacks in the blue channel with the Default method using the thresholding tool in Fiji. Binary cleanup was performed image frame by image frame. The cell mask underwent one closing operation and one fill-holes operation, with no dilation or erosion. The nuclear mask was filled and dilated once before subtraction. The final cytoplasm mask was calculated as the cell mask minus the nuclear mask. The mask and corresponding micrograph stacks in the green and red channels were cropped to the cytoplasm-mask boundaries with a 20-pixel margin. Colocalization analysis was performed in three dimensions with Fiji Coloc 2 plugin version 3.0.5. Green signal (LC3) was assigned as channel 1 and red signal (candidate protein) as channel 2. Costes threshold regression was used, with a PSF size of 3 and 10 Costes randomisations.

## Statistical analysis

All statistical analysis was performed in GraphPad Prism version 10. All datasets were assessed for outliers and normality, followed by appropriate statistical analyses, including one-way ANOVA or the Kruskal–Wallis test, with Fisher's least significant difference or uncorrected Dunn's *post hoc* tests applied where appropriate. Values were represented as the mean ± standard error of the mean (SEM), and significance was accepted at *p* < 0.05.

## Results

### Macroautophagy responds dynamically to increasing APP overexpression

A WST-1 cell viability assay was performed to confirm the appropriate BA treatment duration for subsequent western blot and confocal imaging experiments. Consistent with our previous findings (Ntsapi and Loos, 2021), BA treatment induced a progressive and significant reduction in cell viability. Based on these results, 0, 24, and 48 h were selected to represent increasing levels of proteotoxic stress for subsequent experiments.

Western blot analysis was performed to assess the relative protein abundance of APP and LC3 at different levels of APP overexpression (0, 24, 48 h) in the absence and presence of 400 nM BafA1 (+ BafA1) (Figure 1A–C). Our results reveal that APP protein levels were significantly increased at 24 h [2.99 ± 0.41 (*p* < 0.05)] and 48 h BA [3.18 ± 0.27 (*p* < 0.05)] compared to the control [1.00 ± 0.00] (Figure 1A, B). Likewise, APP protein levels were significantly increased at 24 h [3.79 ± 0.55 (*p* < 0.05)] and 48 h [3.94 ± 0.67 (*p* < 0.05)] compared to the control in the presence of BafA1 [1.91 ± 0.40]. However, no significant differences were detected between the BA-only and BafA1-treated groups (Figures 1A, B). Figure 1A, C indicates the relative protein abundance of LC3. LC3 levels in the 24 h BA [0.23 ± 0.08] and 48 h BA [1.31 ± 0.24] groups showed minimal change compared to the basal level (C0) [1.00 ± 0.00]. In contrast, LC3 increased significantly in all BafA1-treated groups (Figure 1A, C). A significant increase was observed in the control + BafA1 group [4.16 ± 1.42 (*p* < 0.05)] compared to the control [1.00 ± 0.00]. LC3 levels were also significantly elevated in the 24 h + BafA1 group [8.81 ± 0.75 (*p* < 0.05)] compared to the 24 h group [0.23 ± 0.08] (Figures 1A, C). Similar results were observed for the 48 h + BafA1 group [13.90 ± 1.66 (p < 0.05)] relative to the 48 h group [1.31 ± 0.24]. In order to assess the candidate proteins, i.e., APP, Aβ, a-syn, tau and p-tau, in the context of their recruitment to the autophagy compartment, immunofluorescence was performed (Figure 1D). Upon visual inspection, APP (red) showed a marked increase in abundance at both the 24 h and 48 h BA time points, with an even greater increase observed in the 24 h + BA and 48 h + BafA1 groups, indicated by prominent fluorescent signal APP clusters (Figure 1D). In the 0 h BA group (control), LC3 (green) appeared at a basal level, displaying only few LC3 positive clusters distributed throughout the cytoplasm (Figure 1D). As APP expression increases, LC3′s signal intensity initially decreased at the 24 h BA group compared to the control. By 48 h BA, however, its signal intensity rises noticeably. LC3 remained broadly distributed throughout the cell, but with a weaker signal alongside several distinct, brightly fluorescent clusters. When comparing the 0, 24, 48 h of BA groups, there was a clear increase in overall signal intensity and the number of strongly fluorescent LC3 clusters, i.e., the autophagy compartment. Interestingly, in the presence of BafA1, LC3 signal adopted a slightly different pattern: at 0 h BA + BafA1 and 24 h BA + BafA1, it emerged in more localized hotspots rather than being broadly distributed. However, by 48 h BA + BafA1, LC3 changes again, this time becoming less confined to hotspots and instead appearing in numerous smaller, highly intense clusters distributed throughout the cell (Figure 1D). Upon overlay of the APP and LC3 channels, distinct regions of overlap were observed. Although both APP and LC3 increased markedly with disease severity, their relative degree of colocalization appeared to decrease across the BA-treated groups. However, when comparing the BA treated groups in the absence and presence of BafA1 (24h vs. 24h + BafA1, etc), there was a marked increase in colocalization between APP and LC3.

**Figure 1:**
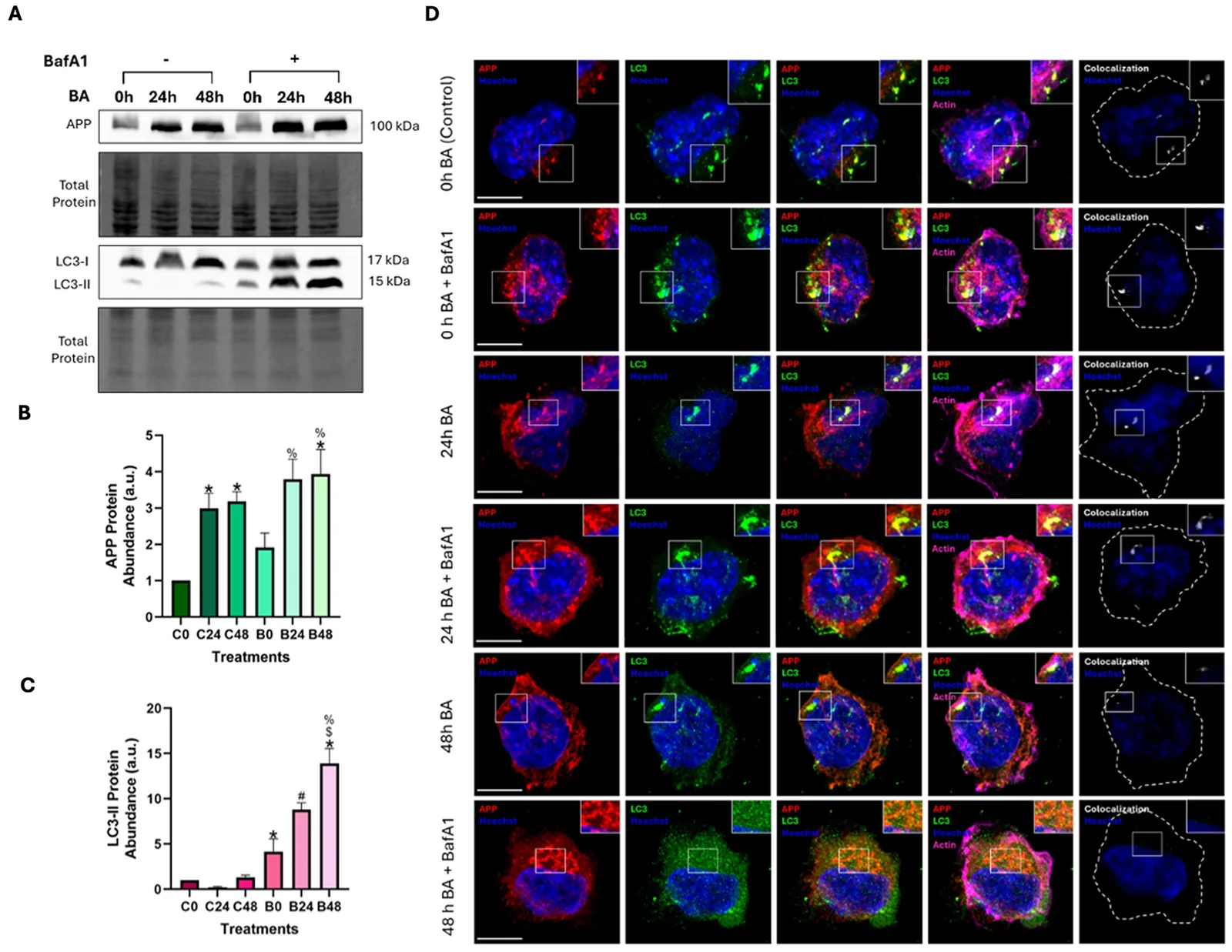
Localization and abundance of APP and LC3 following 24 h and 48 h BA treatment in the presence and absence of BafA. **(A)** Representative western blot analysis and densitometric analysis depicting the levels of **(B)** APP and **(C)** LC3 protein abundance. Representative immunoblots indicate the relative abundance level of APP and LC3 across the various treatment groups: C0: 0 h BA (control); C24: 24 h BA; C48: 48 h BA; B0: 0 h BA + BafA1; B24: 24 h BA + BafA1; B48: 48 h BA + BafA1. Data is expressed as a fold change relative to the control (mean ± SEM; *n* = 3, arbitrary units (a.u.)). *p < 0.05 vs. C0, ^#^*p* < 0.05 vs. C24, ^$^*p* < 0.05 vs. C48, ^%^*p* < 0.05 vs. B0. **(D)** Representative immunofluorescence micrographs illustrating the APP signal distribution across different stages of APP overexpression (0 h BA, 24 h BA, and 48 h BA) as well as the colocalization of APP (red) with LC3B (green) in the presence (+BafA1) and absence of BafA1. Nuclei are counterstained with Hoechst 33342 (blue) and actin filaments are counterstained with actin-phalloidin (magenta). A total of 8-10 cells were acquired. Scale bar: 10 μm.

### Aβ displays limited macroautophagy targeting during APP overexpression

Western blot analysis was employed to assess the relative protein abundance levels of Aβ peptide at different levels of APP overexpression (0, 24, 48 h) in the absence and presence of 400 nM BafA1 (+ BafA1) (Figure 2A, B). Aβ peptide levels were significantly increased at 48 h BA [2.15 ± 0.66 (*p* < 0.05)] compared to the control [1.00 ± 0.00]. Similarly, in the presence of BafA1, Aβ levels were significantly higher at 48 h + BafA1 [2.20 ± 0.17 (*p* < 0.05)] than the control + BafA1 [0.93 ± 0.20]. Additionally, the 48 h +BafA1 group [2.20 ± 0.17 (*p* < 0.05)] was significantly elevated relative to the 24 h + BafA1 group [1.25 ± 0.27]. However, no significant differences were detected between the BA-only- and BafA1-treated groups and their respective time points (Figure 2B). Upon image processing, 3D-micrographs were overlayed and the degree of colocalization was visualized by thresholding and overlapping the Aβ (red) and LC3 (green) channels, with the colocalised voxels displayed in white. Colocalization was further rendered in 3D and displayed with the nucleus to indicate the spatial orientation of the colocalised signal within the cell (Figure 2C). Aβ (red) localization manifests with a modest increase in abundance, with the number of visible clusters increasing as the APP overexpression progresses (Figure 2C). These clusters appeared most pronounced in the presence of BafA1. However, the relative degree of colocalization between Aβ and LC3 (green) remained relatively low across all stages of disease severity in both the absence and presence of BafA1. Interestingly, the degree of colocalisaton appeared most prominent in the control group in the presence of BafA1 (B0 = 0 h +BafA1) (Figure 2C).

**Figure 2:**
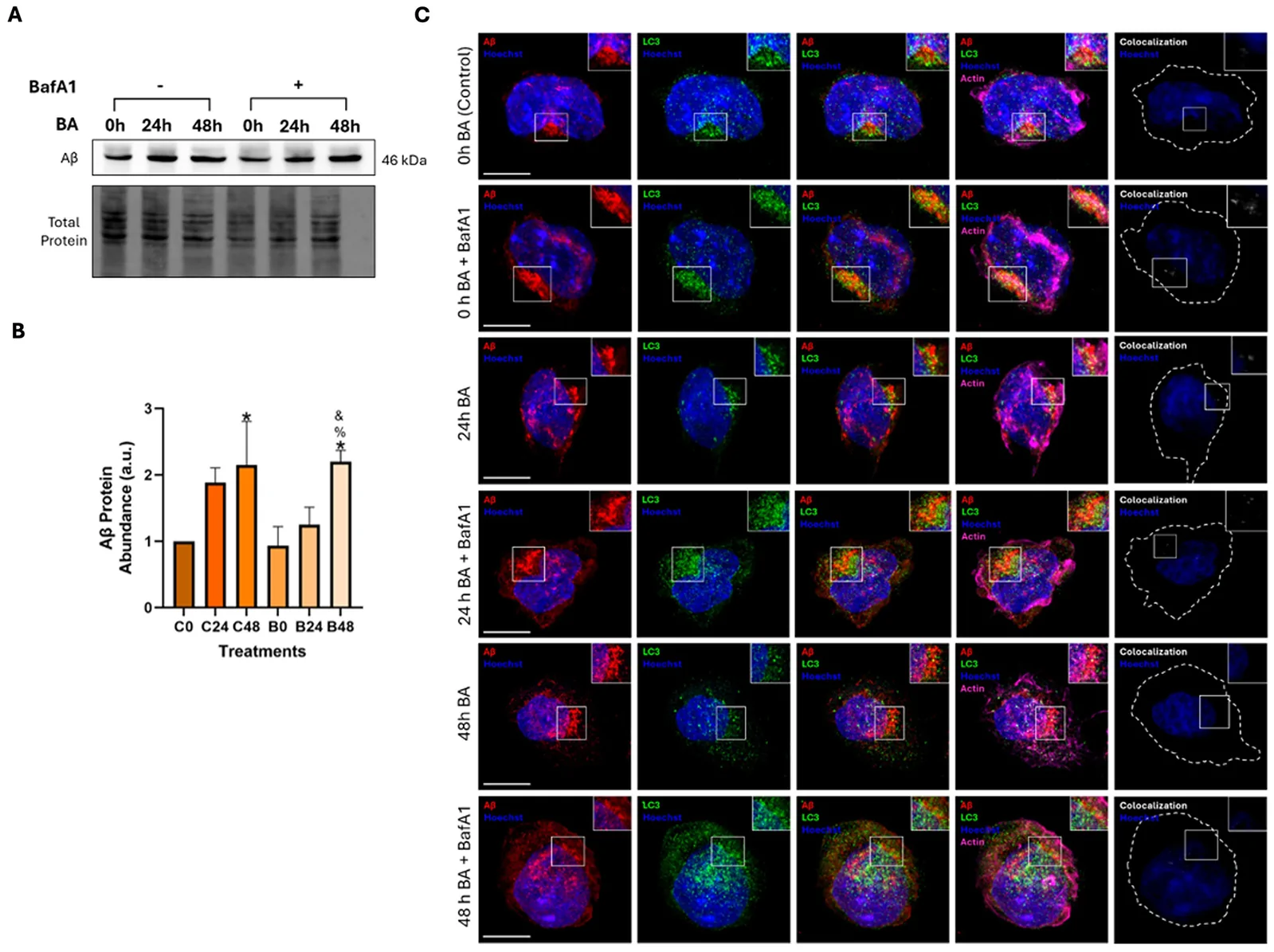
Localization and abundance of Aβ following 24 h and 48 h BA treatment in the presence and absence of BafA. **(A)** Representative western blot and **(B)** densitometric analysis depicting the levels of Aβ protein abundance. Representative immunoblots indicate the relative abundance level of Aβ across the various treatment groups: C0: 0 h BA (control); C24: 24 h BA; C48: 48 h BA; B0: 0 h BA + BafA1; B24: 24 h BA + BafA1; B48: 48 h BA + BafA1. Data is expressed as a fold change relative to the control (mean ± SEM; n = 3, arbitrary units (a.u.)). *p < 0.05 vs. C0, ^%^*p* < 0.05 vs. B0, ^&^*p* < 0.05 vs. B24. **(C)** Representative immunofluorescence micrographs demonstrating the Aβ signal distribution across different stages of APP overexpression (0 h BA, 24 h BA, and 48 h BA) as well as the colocalization of Aβ (red) with LC3B (green) in the presence (+BafA1) and absence of BafA1. Nuclei are counterstained with Hoechst 33342 (blue) and actin filaments are counterstained with actin-phalloidin (magenta). A total of 8-10 cells were acquired. Scale bar: 10 μm.

### α-syn accumulates at advanced stages of APP overexpression with limited macroautophagy targeting

Western blot analysis was employed to assess the relative protein abundance levels of α-syn at different levels of APP overexpression (0, 24, 48 h) in the absence and presence of 400 nM BafA1 (+ BafA1) (Figures 3A, B). Results reveal that the relative protein abundance of α-syn increased significantly at 48 h BA [3.22 ± 1.04 (*p* < 0.05)] relative to 24 h BA [0.78 ± 0.32] (Figures 3A, B). A similar pattern was observed between the 48 h + BafA1 group [2.74 ± 1.17 (*p* < 0.05)] and 24 h + BafA1 group [0.73 ± 0.48]. While α-syn levels appeared higher in the 0 h ± BafA1 group [2.34 ± 1.20] than in the untreated control [1.00 ± 0.00], this difference was not statistically significant. Moreover, no significant differences were observed between the BA-only and BafA1-treated groups at any corresponding treatment time point (Figure 3B), indicating that BafA1 treatment did not significantly alter α-syn abundance under basal conditions. Micrographs reveal that, at 0 and 24 hrs, α-syn levels remained relatively low, with a substantial increase at the 48 h time point in both the absence and presence of BafA1 (Figure 3C). In terms of colocalization, there was a noticeable degree of overlap observed between α-syn and LC3 in 0 h + BafA1 group. However, as the disease severity progressed (24 h and 48 h) the degree of colocalization remained relatively unchanged in both the presence and absence of BafA1 (Figure 3C).

**Figure 3:**
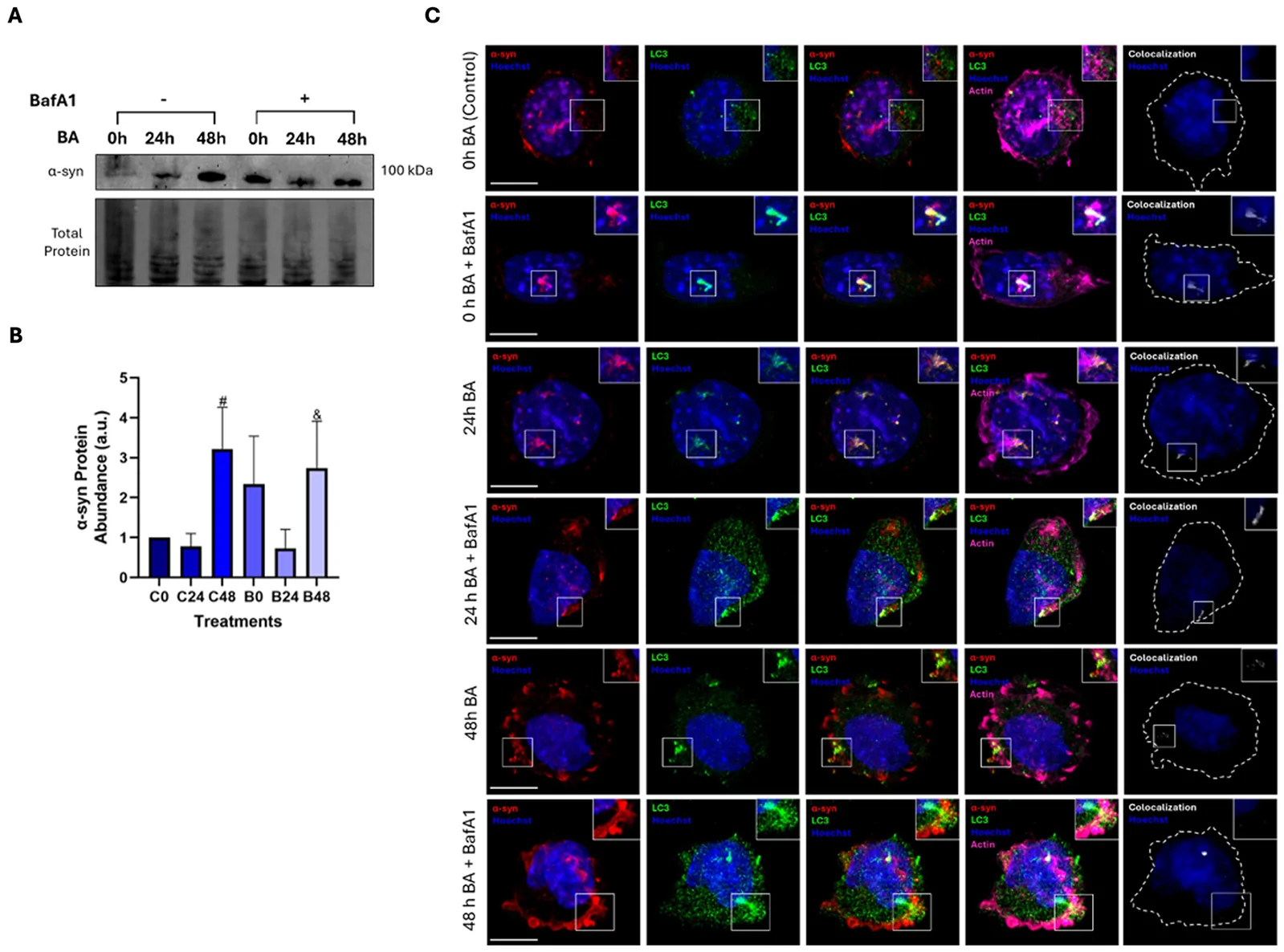
Localization and abundance of α-syn following 24 h and 48 h BA treatment in the presence and absence of BafA. **(A)** Representative western blot and **(B)** densitometric analysis depicting the levels of α-syn protein abundance. Representative immunoblots indicate the relative abundance level of α-syn across the various treatment groups: C0: 0 h BA (control); C24: 24 h BA; C48: 48 h BA; B0: 0 h BA + BafA1; B24: 24 h BA + BafA1; B48: 48 h BA + BafA1. Data is expressed as a fold change relative to the control (mean ± SEM; n = 6, arbitrary units (a.u.)). ^#^*p* < 0.05 vs. C24, ^&^*p* < 0.05 vs. B24. **(C)** Representative immunofluorescence micrographs displaying the α-syn signal distribution across different stages of APP overexpression (0 h BA, 24 h BA, and 48 h BA) as well as the colocalization of α-syn (red) with LC3B (green) in the presence (+BafA1) and absence of BafA1. Nuclei are counterstained with Hoechst 33342 (blue) and actin filaments are counterstained with actin-phalloidin (magenta). A total of 8-10 cells were acquired. Scale bar: 10 μm.

### Tau accumulates with increasing APP overexpression and shows progressive macroautophagy recruitment

Western blot analysis was employed to assess the relative protein abundance levels of tau at different levels of APP overexpression (0, 24, 48 h) in the absence and presence of 400 nM BafA1 (+ BafA1) (Figures 4A, B). Results revealed that the protein levels of tau increased significantly at the 48 h BA time point [19.04 ± 3.69 (*p* < 0.05)] when compared to the control [1.00 ± 0.00] and the 24 h BA time point [5.52 ± 1.63] (Figures 4A, B). In the presence of BafA1, a similar trend was observed: 48h + BafA1 [19.52 ± 2.56 (*p* < 0.05)] was significantly elevated compared to the 0 h + BafA1 [2.25 ± 0.87] and 24 h + BafA1 [8.64 ± 0.70] groups, respectively. Additionally, the 24 h + BafA1 (8.64 ± 0.70 *p* < 0.05) group was also found to be significantly increased relative to the 0 h + BafA1 [2.25 ± 0.87]. No significance was detected between the BA-only and BafA1 treated groups and their respective time points (Figure 4B). Tau abundance gradually increased with disease severity, i.e., APP overexpression, reaching a pronounced peak at the 48 h BA time point. The number of visible clusters also surged as severity progressed (Figure 4C). In terms of the colocalization between tau and LC3, rather than forming distinct hotspots, tau appeared to be distributed across smaller clusters, which was particularly evident in the 48 h + BafA1 group. Overall, the degree of colocalization visually increased as the level of severity increased in both the absence and presence of BafA1(Figure 4C).

**Figure 4:**
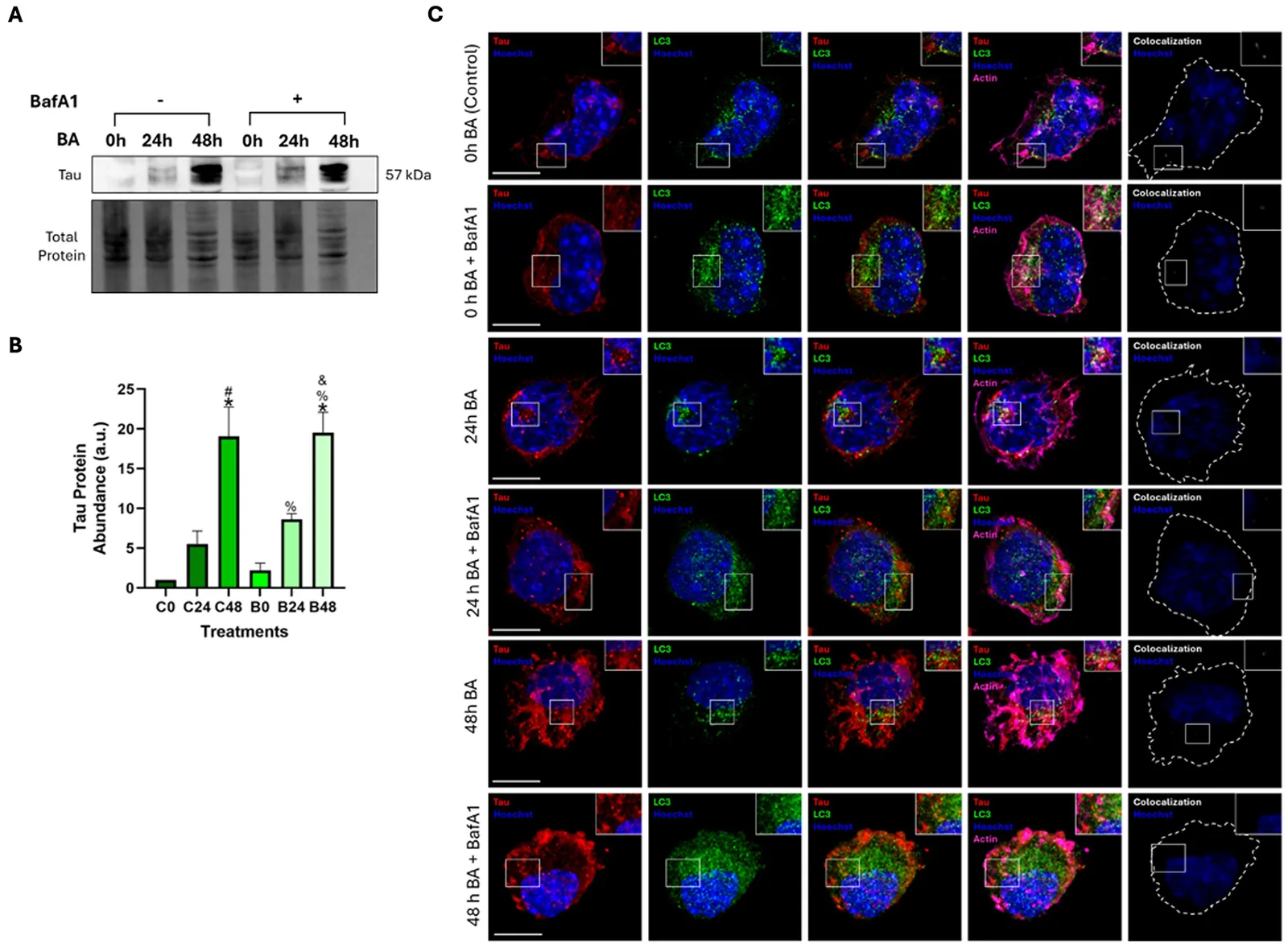
Localization and abundance of tau following 24 h and 48 h BA treatment in the presence and absence of BafA. **(A)** Representative western blot and **(B)** densitometric analysis depicting the levels of tau abundance. Representative immunoblots indicate the relative abundance level of tau across the various treatment groups: C0: 0 h BA (control); C24: 24 h BA; C48: 48 h BA; B0: 0 h BA + BafA1; B24: 24 h BA + BafA1; B48: 48 h BA + BafA1. Data is expressed as a fold change relative to the control (mean ± SEM; n = 3, arbitrary units (a.u.)). *p < 0.05 vs. C0, ^#^*p* < 0.05 vs. C24, ^%^*p* < 0.05 vs. B0, ^&^*p* < 0.05 vs. B24. **(C)** Representative immunofluorescence micrographs representing the tau signal distribution across different stages of APP overexpression (0 h BA, 24 h BA, and 48 h BA) as well as the colocalization of tau (red) with LC3B (green) in the presence (+BafA1) and absence of BafA1. Nuclei are counterstained with Hoechst 33342 (blue) and actin filaments are counterstained with actin-phalloidin (magenta) A total of 8-10 cells were acquired. Scale bar: 10 μm.

### P-tau accumulates with increasing APP overexpression and is strongly recruited to the macroautophagy compartment

Western blot analysis was employed to assess the relative protein abundance levels of p-tau at different levels of APP overexpression (0, 24, 48 h) in the absence and presence of 400 nM BafA1 (+ BafA1) (Figures 5A, B). P-tau significantly increased at the 48 h BA time point [40.87 ± 11.47 (*p* < 0.05)] when compared to the control [1.00 ± 0.00] and the 24 h BA time point [19.42 ± 7.31] (Figures 5A, B). In the presence of BafA1, a similar pattern was observed as 48h + BafA1 [63.53 ± 23.50 (p < 0.05)] was significantly higher than both 0 h + BafA1 [1.54 ± 0.02] and 24 h + BafA1 [29.62 ± 10.85] groups. Furthermore, the 24 h + BafA1 [29.62 ± 10.85 (*p* < 0.05)] group was also found to be significantly increased in p-tau compared to the 0 h + BafA1 [1.54 ± 0.02]. No significance was found between the BA-only and BafA1-treated groups and their respective time points (Figures 5A, B).

**Figure 5:**
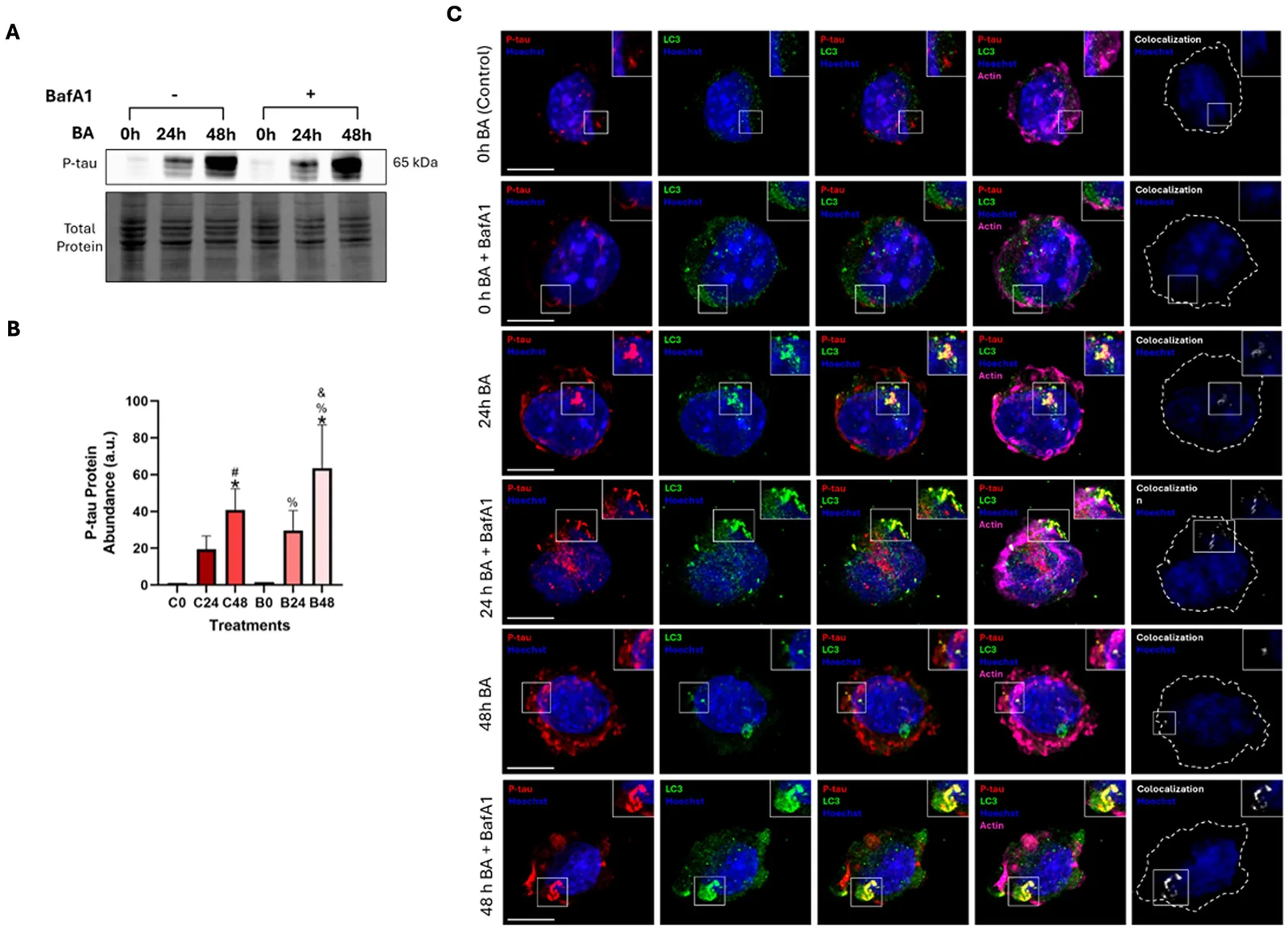
Localization and abundance of p-tau following 24 h and 48 h BA treatment in the presence and absence of BafA. **(A)** Representative western blot and **(B)** densitometric analysis depicting the levels of p-tau abundance. Representative immunoblots indicate the relative abundance level of p-tau across the various treatment groups: C0: 0 h BA (control); C24: 24 h BA; C48: 48 h BA; B0: 0 h BA + BafA1; B24: 24 h BA + BafA1; B48: 48 h BA + BafA1. Data is expressed as a fold change relative to the control (mean ± SEM; *n* = 3, arbitrary units (a.u.)). *p < 0.05 vs. C0, ^#^*p* < 0.05 vs. C24, ^%^*p* < 0.05 vs. B0, ^&^*p* < 0.05 vs. B24. **(C)** Representative immunofluorescence micrographs depicting the p-tau signal distribution across different stages of APP overexpression (0 h BA, 24 h BA, and 48 h BA) as well as the colocalization of p-tau (red) with LC3B (green) in the presence (+BafA1) and absence of BafA1. Nuclei are counterstained with Hoechst 33342 (blue) and actin filaments are counterstained with actin-phalloidin (magenta). A total of 8-10 cells were acquired. Scale bar: 10 μm.

P-tau abundance increased sharply as APP overexpression commenced, corresponding with disease progression, with distinct clusters visible at both the 24 h and 48 h time points (Figure 5C). Although colocalization between p-tau and LC3 was initially minimal at 0 h BA and 0 h BA + BafA1, it increased notably with enhanced disease severity. The highest degree of colocalization was observed at the 24h + BafA1 and 48h + BafA1 time points, respectively (Figure 5C).

### Differential protein responses to APP overexpression and lysosomal inhibition

The relative change in abundance of above candidate proteins was compared across all treatment conditions to identify the most robust responders to APP overexpression and lysosomal inhibition. Protein abundance was normalized to the respective control for each experimental condition, with 24 h (C24) and 48 h (C48) BA-treated groups normalized to the untreated control (C0), and the corresponding BafA1-treated groups (B24 and B48) normalized to the BafA1 control (B0). This analysis was used to determine which proteins exhibited the greatest change in the rate of accumulation across time and treatment conditions (Figures 6A-E).

**Figure 6:**
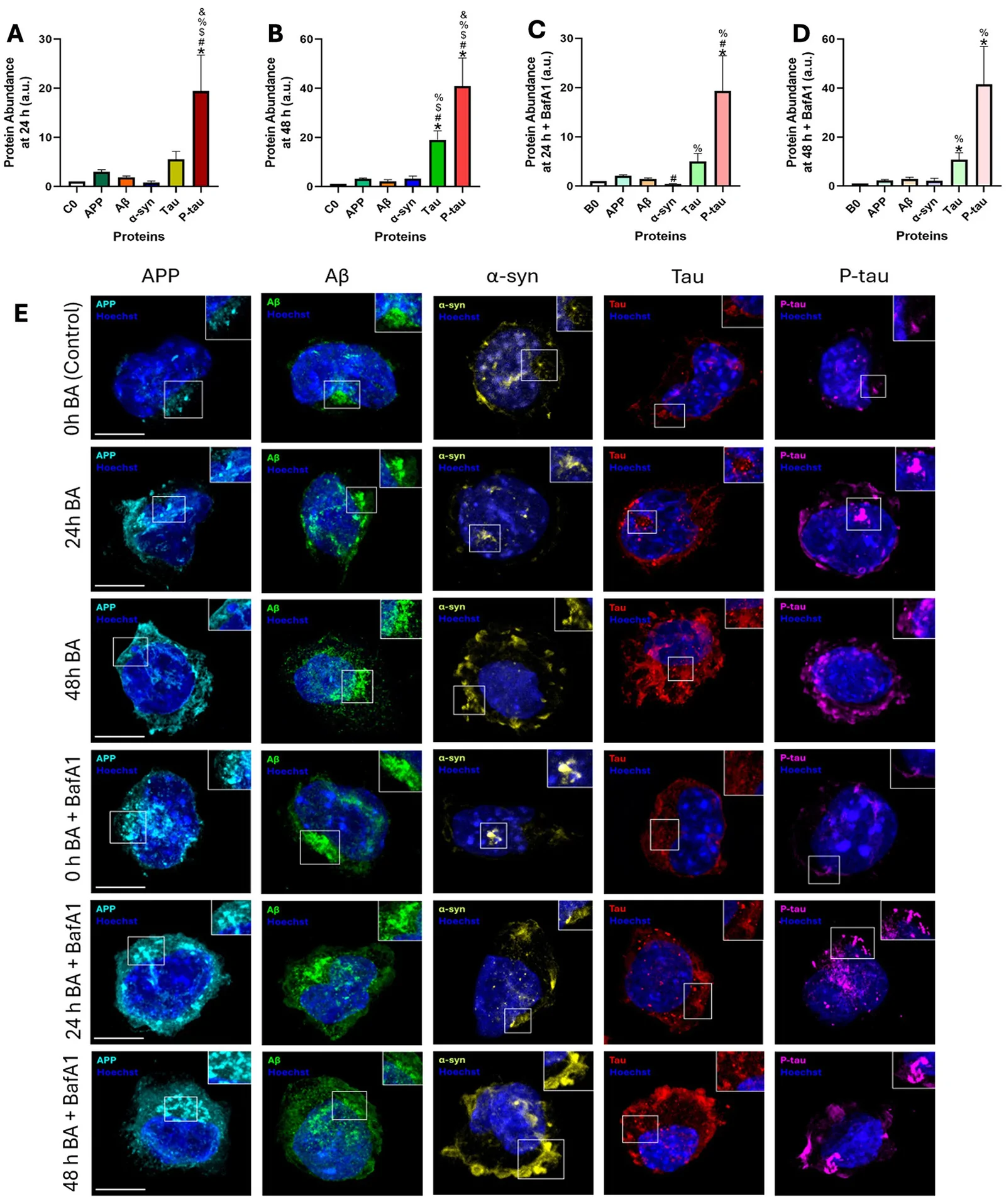
Changes in candidate protein abundance following 24 h and 48 h BA treatment relative to their respective controls in the presence and absence of BafA1. Quantitative and qualitative assessment of candidate protein responses following BA treatment and macroautophagy inhibition. Protein responses following **(A)** 24 h and **(B)** 48 h BA treatment were compared to the control. Protein responses following **(C)** 24 h and **(D**) 48 h BA treatment in the presence of BafA1 were also compared to the control. Data are expressed as fold change relative to the control (mean ± SEM; n = 3, arbitrary units (a.u.)). *p < 0.05 vs. C0, ^#^*p* < 0.05 vs. APP, ^$^*p* < 0.05 vs. Aβ, ^%^*p* < 0.05 vs. α-syn, ^&^*p* < 0.05 vs. tau. **(E)** Representative immunofluorescence micrographs illustrating protein distribution and localization patterns of APP (cyan), Aβ (green), α-syn (yellow), tau (red) and p-tau (magenta) following 24 h and 48 h BA treatment compared to the control, in both the absence and presence of BafA1 (+BafA1). Nuclei were counterstained with Hoechst 33342 (blue). A total of 8–10 cells were imaged. Scale bar: 10 μm.

At 24 h BA, four of the five proteins displayed an increase in abundance relative to the control; however, only p-tau [19.42 ± 7.31 (*p* < 0.05)] was significantly elevated compared to the control [1.00 ± 0.00] and displayed the highest abundance among all proteins, while tau [5.52 ± 1.63] showed a modest but non-significant increase (Figure 6A). Visual analysis of fluorescence micrographs (Figure 6E) supported these findings, with APP, Aβ, tau, and p-tau showing increased signal intensity relative to control, whereas α-syn exhibited reduced intensity and fewer clusters. APP and Aβ formed prominent, strongly fluorescent clusters, tau appeared as smaller cytoplasmic puncta, and p-tau accumulated in fewer but larger hotspots (Figure 6E).

At 48 h BA, p-tau [40.87 ± 11.47 (*p* < 0.05)] remained the most abundant protein relative to control [1.00 ± 0.00], while tau [19.04 ± 3.69 (*p* < 0.05)] also showed a significant increase exceeding all other proteins (Figure 6B). APP [3.18 ± 0.27], Aβ [2.15 ± 0.66], and α-syn [3.22 ± 1.04] displayed modest, non-significant increases. Consistent with this, fluorescence imaging revealed a general increase in protein abundance across all targets, with APP and tau showing the most pronounced increases and widespread cytoplasmic distribution (Figure 6E). Aβ formed numerous distinct clusters, while α-syn accumulated in more defined structural sites rather than diffuse puncta. P-tau exhibited a mixed pattern of elevated intensity and clustered accumulation (Figure 6E). Overall, APP and tau showed the highest signal intensity at this time point.

In the presence of BafA1 at 24 h (B24), p-tau [19.32 ± 7.20 (*p* < 0.05)] was the most abundant protein relative to the BafA1 control (B0) [1.00 ± 0.00], although it was only significantly higher than APP [2.06 ± 0.21] and α-syn [0.35 ± 0.16]. Tau [4.98 ± 1.64 (*p* < 0.05)] was significantly increased compared to α-syn, while α-syn remained significantly lower than APP (Figure 6C). Imaging analysis confirmed that p-tau exhibited the most pronounced change between B0 and B24, transitioning from minimal basal puncta to widespread, strongly fluorescent cytoplasmic clusters (Figure 6E). Tau also increased markedly in both intensity and cluster number, while APP and Aβ remained relatively high with additional clustering. α-syn showed only a moderate increase (Figure 6E). While α-syn immunoreactivity appeared more clustered in the B0 group, this pattern was not accompanied by a statistically significant increase in α-syn abundance compared to the untreated control, suggesting altered intracellular distribution rather than increased protein levels (Figure 6E).

At 48 h + BafA1 (B48), p-tau [41.47 ± 15.60 (*p* < 0.05)] and tau [10.73 ± 2.86 (*p* < 0.05)] were significantly elevated relative to B0 [1.00 ± 0.00], with both proteins exceeding α-syn [2.13 ± 0.98] (Figure 6D). APP [2.20 ± 0.43], Aβ [2.82 ± 0.81], and α-syn showed modest, non-significant increases. Similarly, fluorescence imaging demonstrated increased clustering and intensity across all proteins, with p-tau showing the strongest accumulation and most extensive cluster formation (Figure 6E). Tau displayed both large and small cytoplasmic clusters, APP showed fewer but highly intense clusters, and Aβ formed numerous small dispersed puncta. α-syn exhibited a distinct structured pattern of accumulation with increased intensity (Figure 6E).

Taken together, across both BA and BafA1 conditions, p-tau and tau consistently displayed the most pronounced increase in abundance, while APP and Aβ revealed a more moderate, cluster-associated increase, with α-syn showing a comparatively low and more structurally distinct accumulation pattern.

### Differential association of candidate proteins with LC3 during progressive APP overexpression

To assess the degree of colocalization between LC3 and each target protein, Manders' colocalization coefficients were determined. Manders 1 (M1) represents the proportion of total LC3 signal colocalized with the target protein, i.e. the magnitude of the available autophagy compartment colocalizing with the repective protein to be degraded, whereas Manders 2 (M2) represents the proportion of the total target protein colocalized with LC3, i.e., the magnitude of the overall available proteinaceous cargo colocalizing with the autophsgosomes.

At basal conditions, LC3 exhibited strongest colocalization with APP [0.43 ± 0.07] and Aβ [0.43 ± 0.02], whereas α-syn [0.29 ± 0.06] showed the lowest colocalization with LC3 and was significantly lower than Aβ [0.43 ± 0.02 (*p* < 0.05)] (Figure 7A). When considering the proportion of total target protein colocalized with LC3 (Manders 2), APP [0.33 ± 0.02] remained the most colocalized protein, while Aβ [0.11 ± 0.02 (*p* < 0.05)], α-syn [0.12 ± 0.03 (*p* < 0.05)], and p-tau [0.18 ± 0.05 (p < 0.05)] were significantly less colocalized with LC3 than APP (Figure 7B).

**Figure 7:**
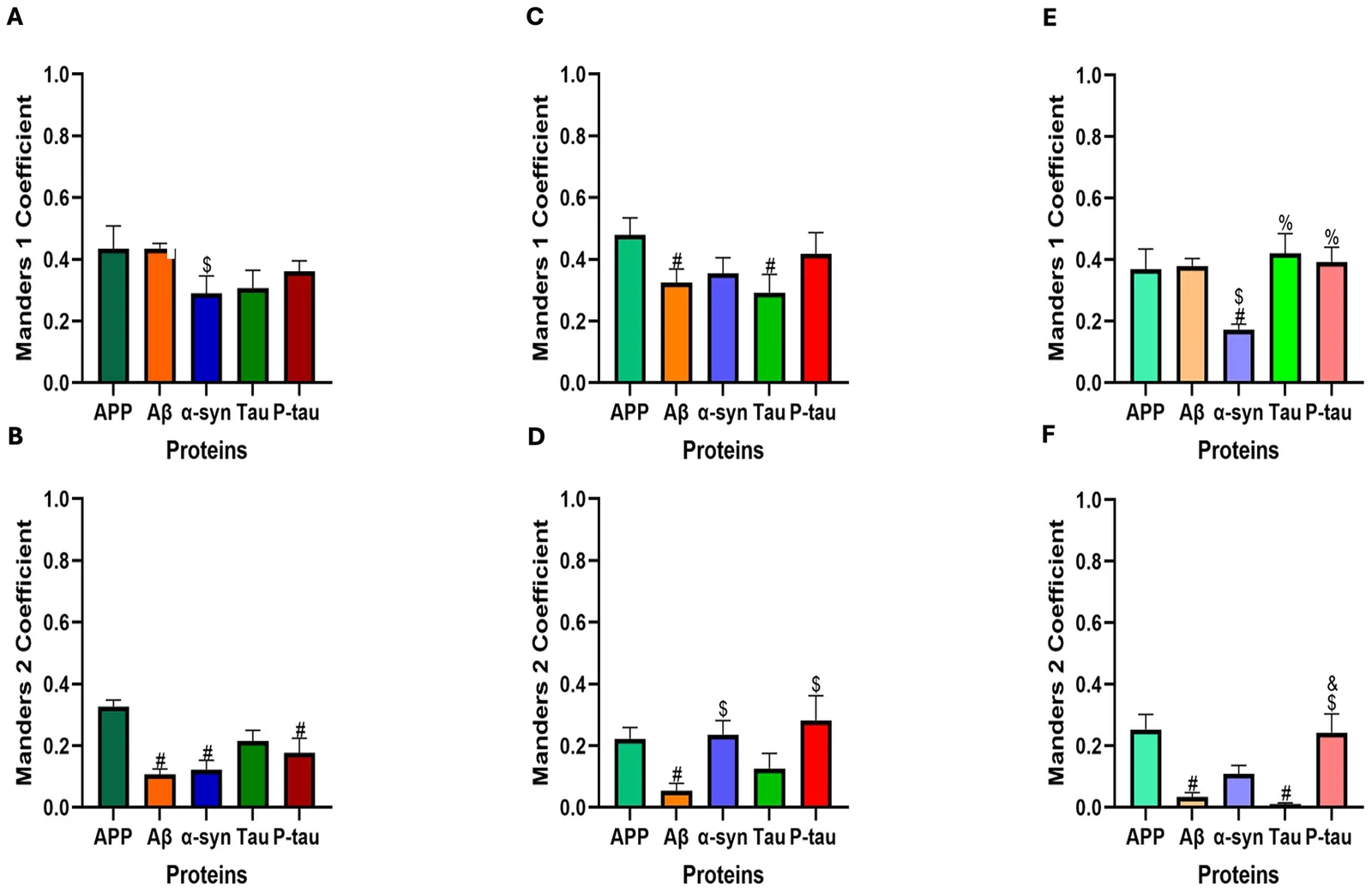
Colocalization analysis of LC3 and candidate proteins using Manders‘ colocalization coefficients following 24 h and 48 h BA treatment. Manders 1 (M1) represents the fraction of total LC3 signal, i.e. the autophagy compartment, colocalized with the respective cargo protein of interest, whereas Manders 2 (M2) represents the fraction of the total cargo protein colocalized with LC3. **(A)** M1 under control conditions. **(B)** M2 under control conditions. **(C)** M1 following 24 h BA treatment. **(D)** M2 following 24 h BA treatment. **(E)** M1 following 48 h BA treatment. **(F)** M2 following 48 h BA treatment. Data are presented as Manders' colocalization coefficients (mean ± SEM; 14–20 cells analyzed). ^#^*p* < 0.05 vs. APP, ^$^p < 0.05 vs. Aβ, ^%^*p* < 0.05 vs. α-syn, ^&^ *p* < 0.05 vs. tau.

At 24 h, APP [0.48 ± 0.05] remained highly colocalized with LC3, whereas Aβ [0.32 ± 0.04(*p* < 0.05)] and tau [0.29 ± 0.06 (*p* < 0.05)] were both significantly less colocalized than APP [0.48 ± 0.05] (Figure 7C). Compared to basal conditions (Figure 7A), α-syn [0.35 ± 0.05] demonstrated a marked increase in its colocalization with LC3, while p-tau [0.42 ± 0.07] also showed a modest increase. In terms of the proportion of total target protein colocalized with LC3, APP [0.22 ± 0.04] decreased slightly compared to basal conditions (Figure 7D vs. Figure 7B). In contrast, α-syn [0.24 ± 0.05] and p-tau [0.28 ± 0.08] increased and exhibited slightly higher values than APP, although these differences were not statistically significant. Both α-syn [0.24 ± 0.05 (*p* < 0.05)] and p-tau [0.28 ± 0.08 (*p* < 0.05)] were, however, significantly higher than Aβ [0.05 ± 0.02], while Aβ [0.05 ± 0.02 (*p* < 0.05)] decreased markedly and remained significantly lower than APP [0.22 ± 0.04] (Figure 7D).

At 48 h, the proportion of LC3 colocalising with α-syn [0.17 ± 0.02 (*p* < 0.05)] decreased substantially and was significantly lower than both APP [0.37 ± 0.07] and Aβ [0.38 ± 0.02] (Figure 7E). In contrast, p-tau [0.39 ± 0.05 (*p* < 0.05)] remained highly colocalized with LC3 and was significantly higher than both α-syn [0.17 ± 0.02] and tau [0.42 ± 0.06]. When assessing the proportion of total target protein colocalized with LC3 (Figure 7F), Aβ [0.03 ± 0.01 (*p* < 0.05)] and tau [0.03 ± 0.02 (*p* < 0.05)] were significantly lower than APP [0.25 ± 0.05], whereas p-tau [0.25 ± 0.06 (*p* < 0.05)] was significantly higher than both Aβ [0.03 ± 0.01] and tau [0.03 ± 0.02]. Compared to 24 h (Figure 7D), α-syn [0.11 ± 0.03] showed a marked reduction in its colocalization with LC3 at 48 h (Figure 7F) and exhibited lower values than both APP [0.25 ± 0.05] and p-tau [0.25 ± 0.06], although these differences did not reach statistical significance.

## Discussion

Macroautophagy (MA) plays a critical role in the degradation of proteins implicated in neurodegenerative diseases, including APP, Aβ, tau, p-tau, and α-synuclein (Webb et al., 2003; Nixon, 2007; Hamano et al., 2021). However, the relative dependence of these proteins on autophagic clearance remains poorly understood. Given emerging evidence linking α-synuclein to Alzheimer's disease pathology (Shim et al., 2022; Franzmeier et al., 2025), this study investigated macroautophagy-mediated clearance and potential differential cargo engagement under APP overexpression-induced proteotoxic stress.

### Macroautophagy engagement during APP overexpression

Macroautophagy was robustly activated in response to APP overexpression, as indicated by increased LC3 levels and autophagic structures (Figure 1). This sustained response across disease stages is consistent with previous reports in both N2a-APP systems and AD models (Joshi et al., 2015; Ntsapi and Loos, 2021; Jin et al., 2022), supporting MA as an early and, importantly, persistent adaptive response to proteotoxic stress, with autophagy flux remaining elevated.

### APP and Aβ: differential engagement with MA

APP levels increased progressively with molecular pathology and were further elevated following MA inhibition, indicating that APP is, at least in part, cleared via MA (Figure 1). Immunofluorescence (Figure 1) and subsequent colocalization analysis (Figure 7) demonstrated strong APP–LC3 colocalization under basal conditions, which declined only modestly with increasing APP-induced proteotoxicity, indicating that APP remained consistently associated with LC3 despite escalating proteotoxic stress. This aligns with evidence suggestive of APP being a MA substrate whose degradation is sensitive to autophagic flux (Jaeger et al., 2010; Tian et al., 2011). In contrast however, Aβ showed only modest accumulation and was not significantly affected by MA inhibition (Figure 2), suggesting limited intracellular retention under these conditions and potentially greater reliance of alternative clearance routes. Consistent with this, Aβ exhibited a distinct colocalization profile, with reduced LC3 association at 24 h followed limited recovery at 48 h (Figure 7), indicating relatively limited engagement with the macroautophagy machinery during disease progression. Together with the western blot and immunofluorescence-based data, these observations support the possibility that intracellular Aβ is cleared through mechanisms other than macroautophagy, including release into the space. This interpretation is supported by recent evidence demonstrating that impaired or altered MA can redirect Aβ toward extracellular secretion pathways, (Atya et al., 2024), consistent with APP membrane processing and extracellular release of Aβ (Chen et al., 2017). As the present study focused exclusively on intracellular Aβ, future studies comparing intracellular and extracellular Aβ levels, for example by quantifying secreted Aβ using ELISA (Nilsson et al., 2013), would assist in determining whether Aβ is indeed preferentially rerouted for extracellular release rather than accumulating intracellularly during progressive proteotoxic stress. In addition, differences in soluble vs. insoluble Aβ pools (Koss et al., 2018) may contribute to reduced detection of intracellular changes. This deserves further study, to better map the relationship between APP and Aβ proteinaceous fate.

### α-syn: secondary response with limited MA dependence

α-Syn exhibited a delayed and very distinct, non-linear response to APP overexpression, with modest accumulation at later stages, with, to our surprise, limited sensitivity to MA inhibition (Figure 3). This strongly suggests that α-syn is not primarily dependent on MA in this context and is likely buffered by alternative pathways such as the ubiquitin–proteasome system and chaperone-mediated autophagy (Birnbaum and Yue, 2025). Of note, colocalization analysis demonstrated a marked increase in α-syn association with LC3 at 24 h, followed by a substantial decline at 48 h (Figure 7), supporting the protein abundance data. This transient recruitment suggests that α-syn is initially recognized by the macroautophagic machinery during the early cellular stress response but becomes a lower-priority substrate as pathology progresses. The subsequent reduction in LC3 association, together with the limited accumulation observed following MA inhibition, further supports the notion that α-syn degradation is progressively compensated by alternative proteolytic pathways. Consequently, the late-stage increase may reflect indirect proteotoxic stress rather than direct APP-driven regulation, consistent with the known pathological interactions of α-syn with Aβ and tau (Badiola et al., 2011). Collectively, these findings suggest that α-syn represents an early, but not sustained, macroautophagic target during progressive proteotoxic stress.

### Tau: accumulation driven by indirect APP toxicity

Tau levels increased markedly with APP overexpression but showed only limited sensitivity to MA inhibition (Figure 4), likely reflecting its ability to be cleared via multiple non-autophagic pathways in its wild-type form, including the proteasome and CMA (Gorantla and Chinnathambi, 2021). Of note, unlike direct tau-overexpression models where MA strongly regulates tau burden (Hamano et al., 2008), the indirect nature of APP-driven pathology in the present model likely delays or attenuates MA dependence. Consistent with this notion, colocalization analysis demonstrated that, although LC3 remained associated with tau throughout disease progression, the proportion of the total tau pool associated with LC3 declined at the later stages (Figure 7). This suggests that macroautophagic recruitment does not increase proportionally with tau accumulation and may instead reflect redistribution of tau toward alternative degradation pathways, such as CMA or the UPS, or the accumulation of tau species that are less accessible to the macroautophagy machinery. While these findings are consistent with previous reports that MA is less efficient at clearing larger or aggregated tau species (Guo et al., 2016), further investigation is required to determine how progressive proteotoxic stress influences tau pathway selection in the present model.

### p-tau: strongest and most consistent MA-associated responding protein

To our surprise, p-tau showed the most pronounced and consistent accumulation across disease stages and was the protein most strongly associated with MA engagement (Figure 5). It is plausible that the PHF-1 epitope (Ser396/Ser404) reflects later-stage, fibrillar tau pathology (Su et al., 1994), suggesting that p-tau accumulation may in this model represent a more advanced pathological state compared to earlier phosphorylation events. Indeed, colocalization analysis demonstrated sustained and progressively elevated LC3 association throughout disease progression (Figure 7), supporting the preferential recruitment of p-tau by macroautophagy during increasing proteotoxic stress. In contrast to total tau, the continued high level of p-tau colocalization with LC3 suggests that phosphorylated tau remains a consistently highly engaging macroautophagic substrate despite advancing pathology. Although in agreement with studies showing MA-dependent regulation of PHF-1–positive tau species (Piras et al., 2016; Lopez et al., 2017) these findings suggest a distinct positioning of p-tau as the most sensitive and consistently engaged substrate within the MA pathway under APP-driven stress, which has not been described previously.

### Integrated model of differential cargo engagement under APP stress

Comparative analysis across the candidate proteins revealed a clear temporal and hierarchical pattern of vulnerability (Figure 6). At early disease stages, p-tau emerged as the most responsive species, followed by tau, APP, Aβ, and α-syn. This pattern was largely preserved under MA inhibition, although differences between proteins became less pronounced. At later stages, tau accumulation became more pronounced, while α-syn increased modestly, suggesting a secondary response to sustained proteotoxic stress. Aβ changes remained comparatively limited across conditions, indicating distinct handling from APP and other aggregation-prone proteins. This progressive pattern is consistent with broader proteomic and *in vivo* studies reporting early disruption of proteostasis networks followed by stage-dependent aggregation cascades (Metaxas et al., 2019; Askenazi et al., 2023), albeit the present study revealing similar recruitment and degradation profiles *in vitro*, when purely driven through APP-overexpression. The persistence of p-tau across all stages, alongside its consistent association with MA engagement, suggests that its phosphorylation status may be a key determinant of autophagic targeting efficiency. This deserves further study.

## Summary and conclusion

This study investigated macroautophagy-mediated processing of key Alzheimer's disease–associated proteins under APP overexpression-induced proteotoxic stress. Our findings demonstrate that APP, Aβ, tau, p-tau, and α-syn exhibit distinct and non-uniform responses to both severity of molecular pathology and dependency on autophagy clearance, indicating selective engagement with the autophagy machinery rather than non-selective degradation. p-Tau emerged as the most consistently elevated and MA-responsive proteinaceous cargo species, followed by APP and α-syn, while Aβ showed limited intracellular engagement with MA and tau displayed a substantially weaker response likely involving alternative degradation pathways. Collectively, these results support a hierarchical model of molecular autophagy machinery engagement in which protein handling is dynamically regulated during APP-driven stress, with implications for stage- and cargo-specific therapeutic targeting of autophagy in Alzheimer's disease (Figure 8). Future work may focus on validating and quantifying the observed differential cargo engagement by expanding the model to include specific cargo receptors, including sequestosome-1/p62, NBR1 or optineurin. Ineed, not only autophagy machinery activity and protein cargo degradation rate, but also specific receptor recruitment profiles and degradation dynamics require consideration (De Wet et al., 2021). Mechanistic studies may determine whether the observed hierarchy is preserved under enhanced macroautophagy activity and how chaperone-mediated autophagy (CMA) and the ubiquitin–proteasome system (UPS) contribute to compensatory clearance (Peker and Gozuacik, 2020; Assaye and Gizaw, 2022). This may include pathway-specific markers for CMA (Patel and Cuervo, 2015) and ubiquitination profiling and will assist in better dissecting the degree of independence from MA. Given the point-of departure approach of this rather reductionist model of proteotoxic stress, and also the limitations using LC3 staining in the context of BafA1, probes that enable pool size analysis of autophagosomes, autolysosomes and lysosomes using LC3-tandem constructs, (Kimura et al., 2007), lysosome markers such as LAMP1/2 or a direct readout of autophagy flux (Yim et al., 2022) may be considered and validated in more complex neuronal model systems, including iPSC-neurons. Resolving both, soluble and insoluble protein fractions and incorporating extracellular Aβ measurements and organelle-specific fractionation may further improve interpretation of protein dynamics (Devi and Anandatheerthavarada, 2010; Nilsson et al., 2013; Koss et al., 2018; Wang et al., 2024). Finally, while examining direct interactions and colocalization among APP, Aβ, tau, p-tau, and α-syn, and extending analyses to *in vivo* or post-mortem tissue, will be essential to confirm the hierarchical autophagy response in human pathology (Shim et al., 2022; Neubauer et al., 2025).

**Figure 8:**
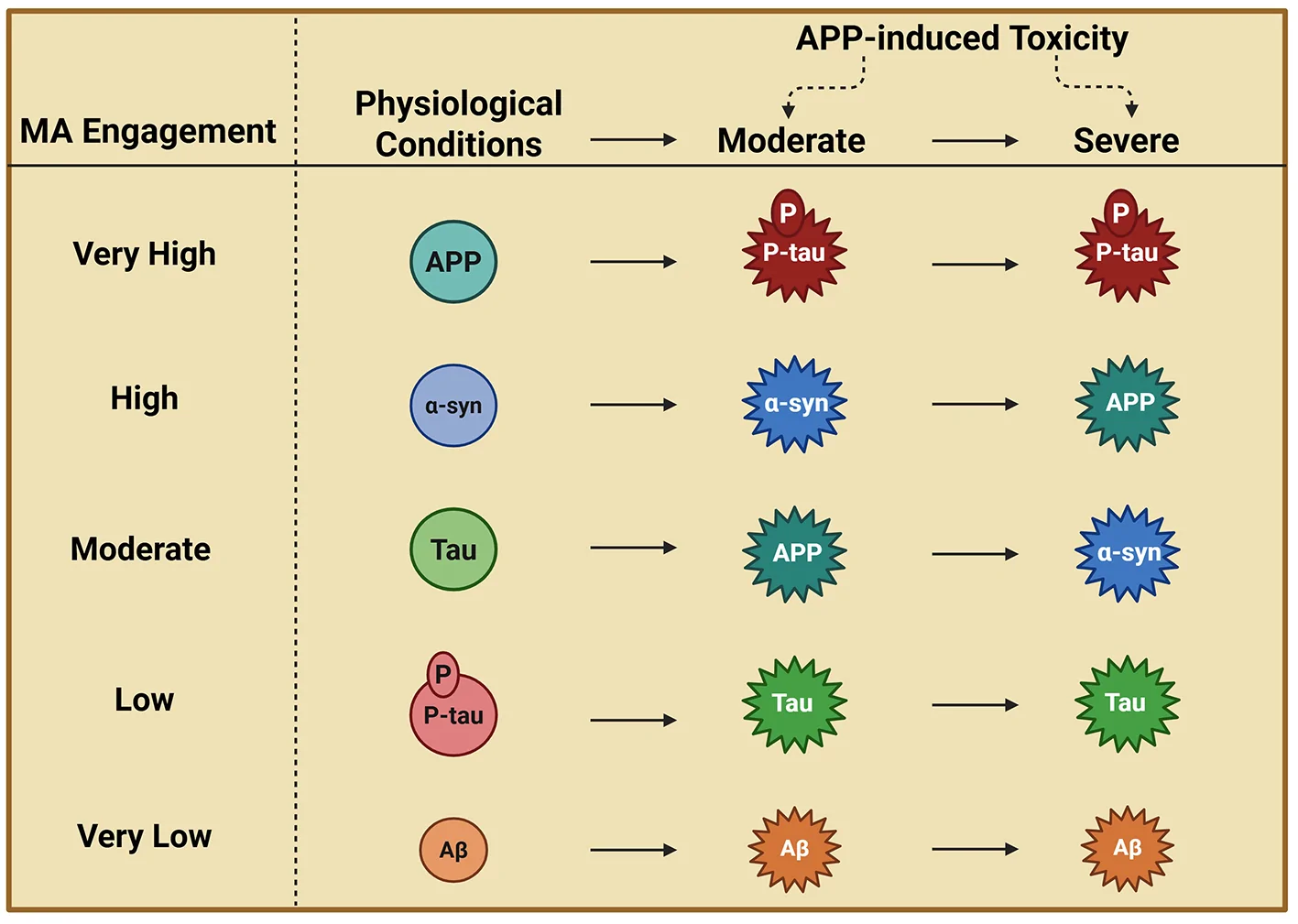
Differential cargo engagement with the macroautophagy compartment during APP overexpression. Schematic summary highlighting key findings. Created in BioRender.

## Data Availability

The original contributions presented in the study are included in the article/supplementary material, further inquiries can be directed to the corresponding author/s.
